# Determination of the Tensile Properties and Biodegradability of Cornstarch-Based Biopolymers Plasticized with Sorbitol and Glycerol

**DOI:** 10.3390/polym13213709

**Published:** 2021-10-27

**Authors:** M. M. Harussani, S. M. Sapuan, A. H. M. Firdaus, Yaser A. El-Badry, Enas E. Hussein, Zeinhom M. El-Bahy

**Affiliations:** 1 Advanced Engineering Materials and Composites Research Centre (AEMC), Department of Mechanical and Manufacturing Engineering, Universiti Putra Malaysia (UPM), Serdang 43400, Selangor, Malaysia; mmharussani17@gmail.com (M.M.H.); muhdfirdaus0701@gmail.com (A.H.M.F.); 2 Laboratory of Biocomposite Technology, Institute of Tropical Forestry and Forest Products, Universiti Putra Malaysia (UPM), Serdang 43400, Selangor, Malaysia; 3 Chemistry Department, Faculty of Science, Taif University, Khurma, P.O. Box 11099, Taif 21944, Saudi Arabia; y.elbadry@tu.edu.sa; 4 National Water Research Center, P.O. Box 74, Shubra El-Kheima 13411, Egypt; enas_el-sayed@nwrc.gov.eg; 5 Department of Chemistry, Faculty of Science, Al-Azhar University, Nasr City, Cairo 11884, Egypt; zeinelbahy@azhar.edu.eg

**Keywords:** cornstarch biopolymers, plasticizers, biodegradable film, physical properties, thermal properties, tensile properties

## Abstract

In this study, the effects of various quantities of sorbitol and glycerol plasticizers (0%, 30%, 45%, and 60%) on cornstarch-based film were examined to develop a novel polymer for usage with biodegradable materials. The film was prepared using the casting process. According to the test findings, the application of the plasticizer concentrations affected the thickness, moisture content, and water absorption of the film. When plasticizer concentrations were increased to 60%, the tensile stress and Young’s modulus of plasticized films dropped regardless of plasticizer type. However, the thin film with addition of 30% sorbitol plasticizer demonstrated a steady value of Young’s modulus (60.17 MPa) with an increase in tensile strength (13.61 MPa) of 46%, while the lowest combination of tensile strength and Young’s modulus is the film that was plasticized with 60% glycerol, with 2.33 MPa and 16.23 MPa, respectively. In summary, the properties and performance of cornstarch-based film were greatly influenced by plasticizer types and concentrations. The finest set of features in this research appeared in the film plasticized with 30% sorbitol, which achieved the best mechanical properties for food packaging applications.

## 1. Introduction

In contrast to biodegradable material, natural chemicals in any accessible human ecosystem are unable to easily decompose. An outstanding example is synthetic plastics, used as the main material in various goods and equipment in engineering, automotive, packaging, and electrical applications [1,2]. The most sophisticated plastics are designed to be resistant to natural decay, remaining on earth for thousands of years without any degradation [3]. The climate, aquatic or land-originated floras and faunas, and humans are all impacted by plastic pollution [4]. There are two methods of solving this problem: recycling and thermal decomposition, including the incineration and pyrolysis approach [5]. A recycling system is extremely costly since the staff and energy used require significant capital costs. Time and resources are needed for the finished product, including plant covering, washing, drying, grinding, and reprocessing as well as chemical disinfection due to the current COVID-19 pandemic [3,6], whereas plastic incineration creates exponentially high quantities of environmental contaminants, including greenhouse gases (CO_2_), ammonia, and arsenic, which lead to detrimental impacts on the public quality of health and climate change.

The growth of biodegradable natural products in many goods in recent decades has globally increased awareness to substitute conventionally non-biodegradable resources. Polysaccharides including natural starch provide several benefits in substituting traditional plastic polymers, including their low cost due to the facile production process, non-toxicity, biodegradability, and availability [7,8]. Starch was used as one of the most essential and efficient naturally occurring hydrocolloids as a food thickener, stabilizer, icing agent, and water protection element [9]. Cornstarch is the largest marketable type of starch. However, the use of natural starch has several drawbacks, which are heat deficiency, sensitivity towards acidity, and failure to meet food processing criteria [10]. In the last decades, technological improvements resulting in greater ease, greenness, and low cost have culminated in considerable involvement in studies related to pollution of chemical modifications and enzyme alteration demands [11].

Starch is well known for its outstanding film and oxygen tolerance qualities and is an effective natural food packaging for plastic polymers. It includes two main elements, amylose and amylopectin. They have different physicochemical properties as they are composed of traces of glucose. Starch food package fillers have been traditionally researched [12]. Currently, researchers plan to broaden the work here by incorporating collagen starch granulates. The development of a network allows the possibility of binding to collagen gel to gelatinized starch through hydrogen [13]. The hydrogen distinctions between functional classes, such as the hydroxide, amylose, and amylopectin starch polymer chains, as well as carbonyl acid/pool-peptide collagen amines can be strengthened by proprietary gelatinization [14]. Nevertheless, extreme interactions between polysaccharides and gelatin have been recorded in multiple protein-dominated models [15]. Starch-related films are thus brittle and hydrophilic, which restricts production and use. Starch may be combined with different synthetic and natural polymers to combat these disadvantages. Forms are found in many layers: regular polyester mixing [16], carbon fiber [17,18], and zinc [19].

Renewable products derived from natural resources are an important alternative to petroleum-based materials for the more environmentally conscious disposal. Such bio-resources, marked by renewability, conservation, biodegradability, and industrial growth, have a major influence on climate, land, and water. Current cellulose residual fibers and plant-based polymers usually have considerable capacity for conformity with environmental conservation criteria [20,21]. A structured strategy for obtaining the appropriate natural biopolymer and manufacturing methods is conducted by researchers in order to reduce the environmental impact during the chemical life cycle. The biomaterial analysis will also continue to take account of local raw materials, including cornstarch in research projects for the replication of technological growth of biodegradable plastics via solution casting method. According to previous studies, the solution casting approach is suitable for the preparation of biocomposite films using a solvent with variable polarities, such as water [22,23] and 1,2-dichloroethane [24].

This research focuses on the development of natural materials as an alternative to conventional plastic composites material. The goal of this research was to extract cornstarch from fresh corn granules and create a biopolymer by using a solution casting process to add various plasticizers. Characterization methods entail mechanical properties and physical properties of the cornstarch with different plasticizers. This study has demonstrated the impact of different concentrations of sorbitol, glycerol, and sorbitol/glycerol plasticizers on cornstarch-dependent thin films’ mechanical and physical characteristics. The specimens obtained were determined by measurements of density, moisture content, and tensile testing.

## 2. Materials and Methods

### 2.1. Materials

Cornstarch has been excluded from the system of fresh ear granules [25,26]. Cornstarch’s chemical structure has been studied through recorded procedures [27]. Cornstarch has a fat content of 0.26%, lipid content of 7.13%, and essential protein content of 7.7%. The content of amylose and amylopectin in starch was calculated in the context of the domestic method (food analytical chemistry) of 24.64 g/100 g and 75.36 g/100 g of previous projects, respectively [28]. Cornstarch particles ranged from polyhedral to spherical and the size (89%) of the dispersed particles was less than 40 μm. The starch moisture content was 10.45 g/100 g, and the density was 1.4029 g/cm^3^. Evergreen Technology & Equipment Malaysia Sdn. Bhd. received plasticizers for sorbitol and glycerol. Virtually all plasticizers are compliant with polymers [29]. The shortcomings in plasticizer form and amount used in polymers are widely recognized in the industry. The available thermal and mechanical characteristics [30], as well as the barrier and rheological properties required, are the selection parameters.

### 2.2. Preparation of Films

Cornstarch films were made using traditional shooting techniques. Filtered 100 mL water containing 10 g pure cornstarch was heated to 85 ± 2 °C for 20 min with continuous thermal baths. This method should have a standardized suspension system. Recommendation research by Ibrahim et al. [31] on sorbitol plasticizer start from 25% to 55% to increase the content of plasticizer. The three forms of plasticizers were then applied to the shaping water individually at 30, 45, or 60% (*w*/*w*, dry starch base). The solvent was injected at the same temperature for another 20 min. The slurry will cool until it is poured into a thermal cup. The casting mixture was dehydrated for 15 h in an air circulation oven at 65 °C. The dehydrated film was obtained and stored a week until the characterization process was finished in plastic bags at room temperature. The film consisted of S30%, S45%, S60% for sorbitol; G30%, G45%, G60% for glycerol; SG30%, SG45%, and SG60% for mixture of sorbitol and glycerol; the control for cornstarch for non-plasticized cornstarch film is as described below in Table 1.

**Figure 1:**
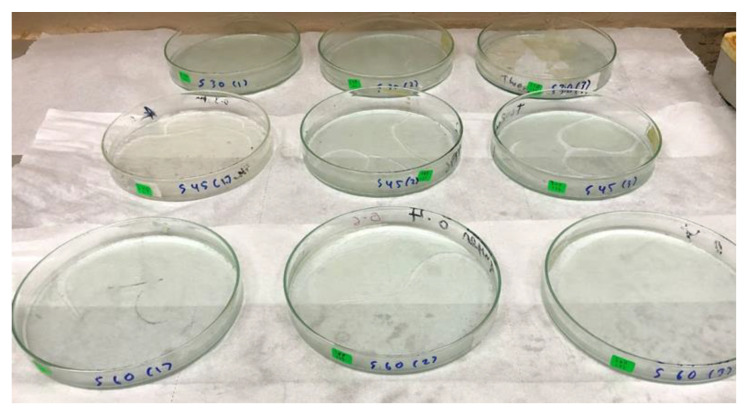
Fabricated cornstarch-based film with different plasticizers.

Cornstarch films are also made utilizing the same formulation process, in which the plasticizer needs to be injected into suspensions of polysaccharides through a certain amount of cornstarch [32,33]. The solution is continuously mixed for a while with a magnetic agitator to achieve an acceptable dispersion. The ideal plasticizer is then poured into a polysaccharide blend with a specified weight/weight ratio. The suspension is then positioned in a water tank through a magnetic agitator at the appropriate temperature and period. The standard suspension is then filled in a petri dish as shown in Figure 1. The dishes are first dried into the air circulation oven. The dry film is then separated into rectangular sections and treated before atmospheric characterization processes [2,5,26,34,35,36,37].

### 2.3. Characterization of Films

#### 2.3.1. Film Thickness and Density

The thickness of the film was evaluated with a ±0.001 inch precision electronic caliper (Mitutoyo-Co, Tokyo, Japan). The mean of five replicated measurements for each film was used to determine the precise thickness of the film. The film density was specifically determined by its volume (V) and mass (g). Where the diameter of each film is determined from the film tests, measurements indicated (10 mm × 30 mm) times the thickness obtained in the previous stage. A gas pycnometer machine was used to measure the density. Thus, density (ρ) is defined as the ratio between the mass (g) and the volume of the specimen (*m*^3^). Equation (1) is used to measure the density.

(1) $$\rho =\frac{m}{V}$$

#### 2.3.2. Film Moisture Content (MC)

The moisture content is defined as the water volume derived from the substance without changing the chemical structure by weight [38]. MC of the film was identified using the method established by Shogren [39]. A common weight film was kept in the oven at 105 °C for 24 h. Shimadzu MOC63u was used to determine the moisture content. For growing specimens shown by percent, weight variations before (*W*_1_) and after (*W*_2_) heat have been added. Moisture content is measured using Equation (2).

(2) $$MC\left(\%\right)=\left[\frac{\left(W_{1}-W_{2}\right)}{W_{1}}\right]\times 100$$

#### 2.3.3. Water Absorption (WA)

A film sample (15 mm × 15 mm) was dried at a 105 °C temperature in a laboratory oven for 3 h and then the film sample was chilled and weighted right away (*m_i_*). The dried sample was submerged in 100 mL of distilled water at room temperature. The sample was immersed in distilled water for 60 min, during which the sample reached equilibrium after around 30 min in water. The sample was pulled from the water after a short immersion period, superficially thawed and weighted with smooth cloth. For the determination of WA, the mass difference between the dry and saturated states in each sample were measured. Equation (3) was used to compute the results of the research.

(3) $$WA\left(\%\right)=\left[\frac{\left(m_{f}-m_{i}\right)}{m_{i}}\right]\times 100$$

#### 2.3.4. Tensile Properties

The universal tensile machine, the 5 kN Instron, was used to test the specimens’ tensile characteristics. It has been used for the usage of the tensile system and camera tests [5,30,40,41]. A 10 × 90 mm film strip was clamped between tensile handles with a 2 mm/min crosshead ratio and a 30 mm grip distance. Differences in the velocity of the crosshead between 0.5 and 10 mm per minute do not impact the composite’s tensile properties. Five studies have been set up to detect tensile strength and elastic modulus. Figures 4–6 shows the 5 kN Instron Tensile Test Machine used in finding the tensile properties.

### 2.4. Statistical Analysis

In SPSS software, the acquired experimental findings of tensile strength were subjected to an analysis of variance (ANOVA). Duncan’s test was used to do a mean comparison at a significance level of 0.05 (*p* ≤ 0.05).

## 3. Results

### 3.1. Film Thickness and Density

**Figure 2:**
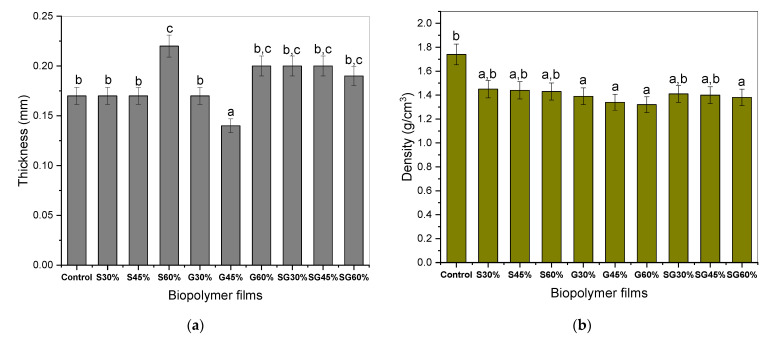
(**a**) Thickness and (**b**) density of cornstarch biopolymer films. ^a,b,c^ Values with different letters in the figures are significantly different (*p* < 0.05).

Figure 2a indicates that with the increase of plasticizer concentration, the thickness of cornstarch-based films dramatically increases. Thus, the films of 60% plasticizers were mostly thicker than the 30–45% plasticizer loading, except for films with sorbitol/glycerol plasticizers. These observations may be relevant in the reconstruction of the polymeric molecular film chain, and the plasticizer position of these findings is important where the higher plasticizer concentration results in a higher weight with the same space and resulted in increased film thickness [42]. In terms of film density, the kind and concentration of plasticizers appeared to have distinct effects.

According to Figure 2b, the increase in sorbitol, glycerol, and mixture of sorbitol and glycerol plasticizer concentration in the cornstarch-based film decreased its density: sorbitol from 1.4460 to 1.4340 g/cm^3^, glycerol from 1.3916 to 1.3193 g/cm^3,^ and mixture of sorbitol and glycerol from 1.4060 to 1.3831 g/cm^3^. This is consistent with the results from previous works [28,40].

### 3.2. Moisture Content

**Figure 3:**
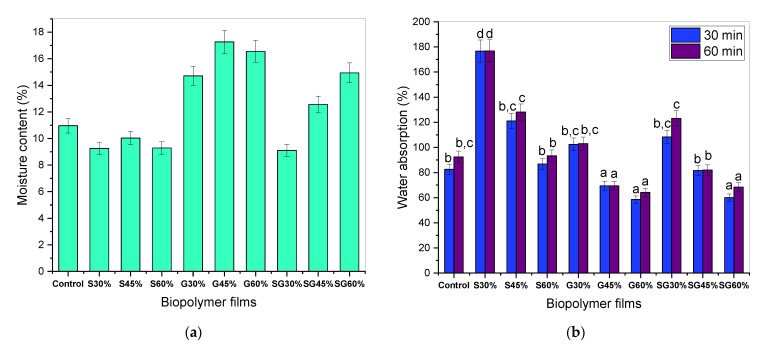
(**a**) Moisture content and (**b**) water absorption of cornstarch biopolymer films. ^a,b,c,d^ Values with different letters in the figures are significantly different (*p* < 0.05).

Regardless of the type of plasticizer, adding 30 to 45% plasticizers improved the moisture content of plasticized films due to their water-retaining capacity [43]. However, the effects of sorbitol and glycerol plasticizers decreased moisture content when it reached 60% loading as shown in Figure 3a. Given the glucose unit similarity of the plasticizer’s molecular structure, the lack of a molecular connection between the plasticizer and the intermolecular biopolymer chains was caused by differences in the moisture content of plasticized films [44]. As a result, the the likelihood of plasticizers interacting with water molecules has increased [45]. When using natural fiber as an advanced fabric for creating modern product materials, the moisture content is a vital consideration. Low moisture content is required because high water levels adversely affect the composite material’s dimensional stability, particularly with regard to mechanical strength, porosity, and water-holding capacity [46].

### 3.3. Water Absorption

One of the most significant constraints of bio-based materials is water absorption. Afterward, the impact of the water absorption rate on the cornstarch-based film was investigated, which was then recorded (Figure 3b). The physical characteristics of bio-based materials, especially water absorption, are noticeable for their effects on tensile strength, porosity forming, dimensional stability, and the final product swelling [46]. In this study, however, measurements of the water absorption rate 30 min and 60 min after samples’ immersion in distilled water were obtained. The cornstarch-based film, using sorbitol-plasticized films, showed a strong tendency to absorb water by 176.80% and decrease from 128.18 to 93.34% in water absorption for 45 and 60% plasticizer content. Related testing showed that the glycerol and mixture of sorbitol and glycerol reduced water absorption, from 103.05 to 64.1% for 30–60% glycerol plasticizer and 123.19 to 68.47% for 30–60% sorbitol/glycerol mixture. The decrease in sorbitol and glycerol absorption rates may be due to the role of plasticizing in the development of heavy interfacial bonds within the system to prevent water entry into the matrix [47]. Table 1 describes the results.

### 3.4. Tensile Properties

**Figure 4:**
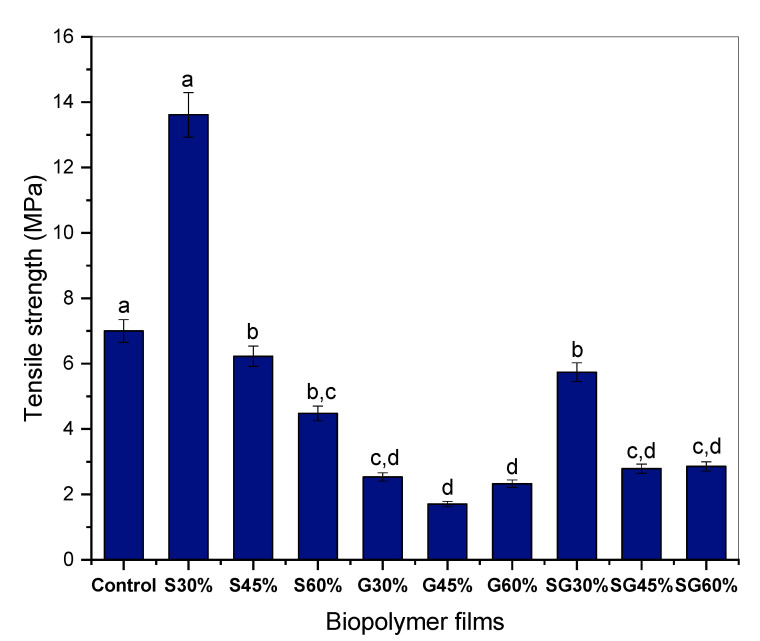
Tensile strength of the cornstarch-based film with different plasticizers. ^a,b,c^ Values with different letters in the figures are significantly different (*p* < 0.05).

**Figure 5:**
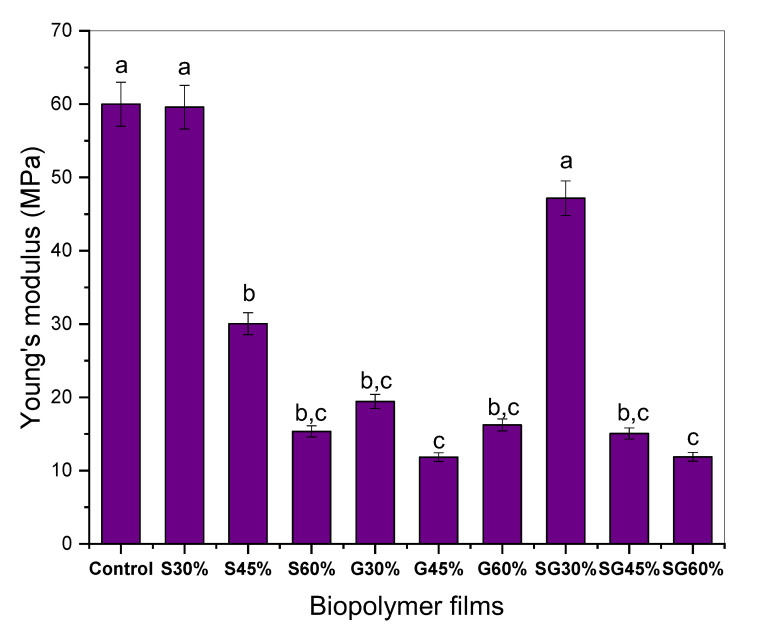
Young’s modulus of the cornstarch-based film with different plasticizers. ^a,b,c^ Values with different letters in the figures are significantly different (*p* < 0.05).

**Figure 6:**
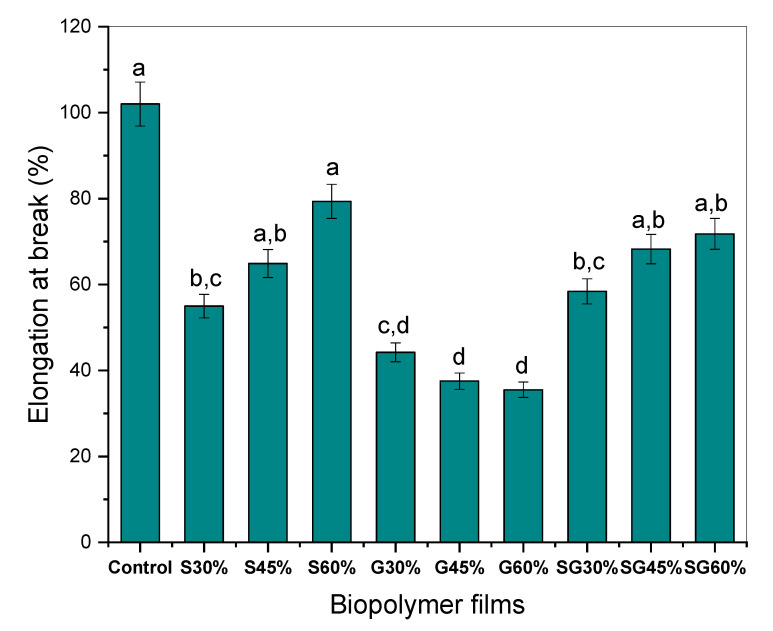
Elongation at break of cornstarch-based film with different plasticizers. ^a,b,c^ Values with different letters in the figures are significantly different (*p* < 0.05).

In particular, on cornstarch-based films, the tensile strength (TS), Young’s modulus (YM), and elongation at break (EB) were assessed using several types of plasticizers. Figure 4, Figure 5 and Figure 6 illustrate the effect of specific plasticizers on the tensile properties of cornstarch-based film. From the findings, the incorporation of cornstarch and the plasticizer of sorbitol resulted in a significant increase in S30% but then a slight decrease for S45% to S60% in TS and YM but increases in EB were showed along the increase in sorbitol addition. From the findings also, the incorporation of cornstarch and glycerol plasticizer resulted in a slight decrease in TS, YM, and EB compared to the control one. Apart from that, the addition of both plasticizers of sorbitol and glycerol resulted in lower TS and YM than the control sample and continued to decrease but increased in elongation at break for SG30%, SG45%, and SG60%. Note that, as for the statistical analysis, a, b, c and d values with different letters in the same column are significantly different (*p* < 0.05), as stated in Figure 4, Figure 5 and Figure 6.

The results show that the maximum tensile strength is S30% with 13.61 MPa, larger than G30% with 2.53 MPa and SG30% with 5.74 MPa. The predicted concept of low plastic strain is related to hydrogen interactions between starch and plasticizer molecules, which are tightly controlled and decrease in quality by decreasing plasticizers [48]. Therefore, the tensile strength for sorbitol-plasticized film then decreased from 13.61 to 4.49 MPa and the mixture of sorbitol and glycerol-plasticized film decreased from 5.74 to 2.26 MPa as plasticizer concentration increased to 45% and 60%. While the plastic films registered the lowest stress levels, they decreased the plasticizer from 2.53 to 2.33 MPa as shown in Figure 4. Many researchers have shown that the tensile strength of starch films declines due to the enhanced plasticizer concentration.

Young’s modulus helps determine the stiffness of materials. The optimal tensile strength of a material is indicated by a greater Young’s modulus value. The figure shows the impact of the cornstarch-based film on the corresponding tensile strength and Young’s modulus for different plasticizers with different concentrations (30–60%). The rise in plasticizer concentrations, from 30 to 60%, shows substantial reductions in Young’s modulus: 60.17–15.35 MPa for sorbitol-plasticized films; 47.17–11.88 MPa for sorbitol/glycerol-plasticized films; and 19.43–16.23 MPa for glycerol-plasticized films. These plasticizers are incorporated into starch chains, promoting the formation of hydrogen bonds between molecules while decreasing the starch matrix’s strong intramolecular attraction. The Young’s modulus for cornstarch-based film is thus smaller and less rigid [29,49].

Based on the elongation at break value of the cornstarch-based film with a plasticizer of sorbitol, glycerol, and a mixture of sorbitol/glycerol, the thin film resulted in a different effect, as can be seen when compared with the tensile strength and Young’s modulus values (Figure 6). As the pressure increases, the elongation at break of the cornstarch-based film with sorbitol and sorbitol/glycerol plasticizer increases. However, the cornstarch-based film with only glycerol plasticizer shows a decrease in elongation at break. This shows the sorbitol plasticizers will produce higher elongation at break while glycerol plasticizer will produce lower elongation at break [33]. The extendibility of the material is calculated from the original length before the breakpoint. With the reconstruction of the composite structure, by strengthening intermolecular links between cornstarch and plasticizer, the film elongation is decreased as the fiber loading is increased. This redesign increases rigidity and durability and reduces chain instability [50].

## 4. Conclusions and Future Outlooks

To solve persistent environmental issues caused by the manufacturing of non-biodegradable polymers, the creation of completely biodegradable films is necessary. Cornstarch is taken from corn plant components, and the properties of cornstarch-based film have also been found to be influenced by different plasticizers that are effectively defined. Furthermore, the intrinsic disadvantages of cornstarch-based films, such as brittleness, inferior mechanical properties, as well as weak water barrier resistance, have restricted their use as a biomaterial. This research intended to improve the functional characteristics of cornstarch. The cornstarch material has also been tested for mechanical and physical properties. Tensile strength, Young’s modulus, elongation at break, density, moisture content, and water absorption of the cornstarch-based film have all been researched to reduce the disadvantages and maximize the efficiency of cornstarch-based film.

The key aim was to determine the effect of various concentrations of sorbitol, glycerol and a sorbitol/glycerol in plasticizers to develop specific polymers for use in biodegradable goods. The solution casting technique was used for the creation of films in this study. Based on the results, the addition of the plasticizers changed the thickness, moisture content, and water absorption. Tensile strength and Young’s modulus dropped when the plasticizer concentration was above 30%, regardless of the plasticizer type. In conclusion, the characteristics and performance of the cornstarch-based film have a considerable impact on the plasticizer kinds and concentrations. The maximum level of mechanical efficiency was reached with film plasticized with 30% sorbitol, with tensile strength and Young’s modulus of 13.61 MPa and 60.17 MPa, respectively. The biopolymer films revealed a novel cornstarch waste resource that is both environmentally beneficial and simple to produce. It also generated new data on the interactions of various plasticizer types and how their concentration affects film capabilities, which might benefit the development of biodegradable materials. For future endeavors, there are several improvements and research that should be worked on:

1. The analysis of hybrid corn compounds should be regarded using certain processing methods for different forms of goods such as extrusion or injection.
2. The research will be carried out to further increase the efficiency through different surface treatment methods of hybrid corn plant composites.
3. Experiments in synthetic polymer films on the potency of natural antimicrobial compounds should be regarded to develop their performance.
4. Experiments should often be taken into consideration to validate the effects of current research using finite element methods.

## Figures and Tables

**Table 1:** Various plasticizer types and concentrations used in cornstarch-based films.

Biopolymer Films	Plasticizers	
	Types	Concentration (%)
**Control**	-	-
**S30%**	Sorbitol	30
**S45%**	Sorbitol	45
**S60%**	Sorbitol	60
**G30%**	Glycerol	30
**G45%**	Glycerol	45
**G60%**	Glycerol	60
**SG30%**	Sorbitol/Glycerol	30
**SG45%**	Sorbitol/Glycerol	45
**SG60%**	Sorbitol/Glycerol	60

## Data Availability

Not applicable.
